# Dual function of GTPBP6 in biogenesis and recycling of human mitochondrial ribosomes

**DOI:** 10.1093/nar/gkaa1132

**Published:** 2020-12-02

**Authors:** Elena Lavdovskaia, Kärt Denks, Franziska Nadler, Emely Steube, Andreas Linden, Henning Urlaub, Marina V Rodnina, Ricarda Richter-Dennerlein

**Affiliations:** Department of Cellular Biochemistry, University Medical Center Goettingen, D-37073 Goettingen, Germany; Cluster of Excellence ‘Multiscale Bioimaging: from Molecular Machines to Networks of Excitable Cells’ (MBExC), University of Goettingen, Goettingen, Germany; Cluster of Excellence ‘Multiscale Bioimaging: from Molecular Machines to Networks of Excitable Cells’ (MBExC), University of Goettingen, Goettingen, Germany; Department of Physical Biochemistry, Max Planck Institute for Biophysical Chemistry, D-37077 Goettingen, Germany; Department of Cellular Biochemistry, University Medical Center Goettingen, D-37073 Goettingen, Germany; Department of Cellular Biochemistry, University Medical Center Goettingen, D-37073 Goettingen, Germany; Bioanalytical Mass Spectrometry Group, Max Planck Institute for Biophysical Chemistry, D-37077 Goettingen, Germany; Bioanalytics, Institute for Clinical Chemistry, University Medical Center Goettingen, D-37073 Goettingen, Germany; Bioanalytical Mass Spectrometry Group, Max Planck Institute for Biophysical Chemistry, D-37077 Goettingen, Germany; Bioanalytics, Institute for Clinical Chemistry, University Medical Center Goettingen, D-37073 Goettingen, Germany; Cluster of Excellence ‘Multiscale Bioimaging: from Molecular Machines to Networks of Excitable Cells’ (MBExC), University of Goettingen, Goettingen, Germany; Department of Physical Biochemistry, Max Planck Institute for Biophysical Chemistry, D-37077 Goettingen, Germany; Department of Cellular Biochemistry, University Medical Center Goettingen, D-37073 Goettingen, Germany; Cluster of Excellence ‘Multiscale Bioimaging: from Molecular Machines to Networks of Excitable Cells’ (MBExC), University of Goettingen, Goettingen, Germany

## Abstract

Translation and ribosome biogenesis in mitochondria require auxiliary factors that ensure rapid and accurate synthesis of mitochondrial proteins. Defects in translation are associated with oxidative phosphorylation deficiency and cause severe human diseases, but the exact roles of mitochondrial translation-associated factors are not known. Here we identify the functions of GTPBP6, a homolog of the bacterial ribosome-recycling factor HflX, in human mitochondria. Similarly to HflX, GTPBP6 facilitates the dissociation of ribosomes *in vitro* and *in vivo*. In contrast to HflX, GTPBP6 is also required for the assembly of mitochondrial ribosomes. GTPBP6 ablation leads to accumulation of late assembly intermediate(s) of the large ribosomal subunit containing ribosome biogenesis factors MTERF4, NSUN4, MALSU1 and the GTPases GTPBP5, GTPBP7 and GTPBP10. Our data show that GTPBP6 has a dual function acting in ribosome recycling and biogenesis. These findings contribute to our understanding of large ribosomal subunit assembly as well as ribosome recycling pathway in mitochondria.

## INTRODUCTION

The biogenesis and the function of the mitochondrial ribosome is of central importance for the fitness and viability of eukaryotic cells, and mutations in genes encoding for mitochondrial ribosomal proteins or translation factors lead to severe human diseases (1,2). Although the human mitochondrial genome (mtDNA) encodes only thirteen polypeptides, all of which are subunits of the oxidative phosphorylation (OXPHOS) system, ∼25% of the mitochondrial proteome is dedicated to the task of mtDNA gene expression (3). More than 12% of the mitochondrial proteome form the translational apparatus to synthesize those thirteen mtDNA-encoded proteins. How the mitochondrial ribosome assembles and which auxiliary factors are required for its function is poorly understood. Even though the human mitochondrial ribosome evolved from a bacterial ancestor, there are significant differences in structure and composition between mitochondrial and bacterial ribosomes. The 55S mitochondrial ribosome is composed of a 28S small ribosomal subunit (mtSSU) with the 12S rRNA and 30 proteins, and a 39S large ribosomal subunit (mtLSU) with the 16S rRNA, the tRNA^Val^ or tRNA^Phe^ in the central protuberance and 52 proteins. The 55S ribosome is a protein-rich particle, with a protein:RNA ratio of 70%:30%, which is reversed compared to the bacterial ribosome (4). Approximately 50% of mitochondrial ribosomal proteins lack a bacterial homolog. Thus, it is likely that the assembly of the mitochondrial ribosome differs from that of the bacterial one and requires additional ancillary biogenesis factors. Although a number of assembly factors have been identified, including RNA helicases, RNA modifying enzymes, chaperones and GTPases, the list of factors required for mitochondrial ribosome biogenesis and function is far from complete (5). Recently, the role of GTPases in human mitochondrial ribosome biogenesis has received particular attention as their loss affects various cellular functions. For example, GTPases ERAL1 and NOA1/C4ORF14 are required for 12S rRNA stability and mtSSU assembly (6–9). GTPBP5, GTPBP7 and GTPBP10 are required for late maturation stages of the mtLSU (10–16): GTPBP7 couples mtLSU assembly to intersubunit bridge formation and governs a quality control checkpoint for mtLSU biogenesis (12). GTPBP10 acts in a complex with other late assembly factors such as NSUN4, MTERF4, MALSU1 and SMCR7L (13–15). GTPBP5, which acts subsequently to GTPBP10, is required for MRM2-catalyzed 16S methylation at position U1369 (16,17). GTPBP5 loss results in an accumulation of a late assembly intermediate containing GTPBP10, MALSU1 and MTERF4, but missing bL36m. Ablation of all these GTPases leads to loss of 55S ribosomes and to mitochondrial translation deficiency.

Human GTPBP6 is a poorly studied member of the translational GTPase family. The function of the factor in human cells is not known. It is homologous to the bacterial GTPase HflX, which is a ribosome-recycling factor facilitating dissociation of 70S ribosomes into subunits (18–20). HflX is not required for *Escherichia coli* viability under laboratory conditions of rapid growth, but becomes essential during heat shock where it helps to recycle damaged ribosomes (18). In addition, HflX may act as an ATP-dependent RNA helicase capable of remodeling damaged rRNA and thus restoring heat-inactivated ribosomes (20).

In the present study, we reveal the function of GTPBP6 in human mitochondria. Elevated levels of GTPBP6 cause an accumulation of mtSSU and mtLSU, suggesting that GTPBP6 facilitates 55S mitochondrial ribosome dissociation *in vivo*. We further validate the activity of GTPBP6 as a ribosome-recycling factor *in vitro* using real-time kinetic assays. GTPBP6 ablation leads to a drastic deficiency in mitochondrial translation due to defects in mitochondrial ribosome assembly resulting in accumulation of mtLSU intermediates at a late maturation stage when MTERF4, NSUN4, MALSU1, GTPBP5, GTPBP7 and GTPBP10 are bound to the mtLSU. Our results suggest that human GTPBP6 has a dual function in facilitating the recycling and biogenesis of mitochondrial ribosomes.

## MATERIALS AND METHODS

### Key reagents

A list of antibodies, oligonucleotides and other materials used within this study is provided in Supplementary Table S1.

### Cell culture

Human embryonic kidney cell line (HEK293-Flp-In T-Rex, HEK293T) was cultured in Dulbecco's modified Eagle's medium (DMEM) supplemented with 10% FBS (Sigma), 2 mM l-glutamine, 1 mM sodium pyruvate, 50 μg/ml uridine, 100 U/ml penicillin and 100 μg/ml streptomycin (GIBCO) under standard cultivation conditions (37°C under 5% CO_2_ humidified atmosphere). Cells were systematically confirmed to be negative for the presence of Mycoplasma by GATC Biotech.

For cell counts cells were grown on standard DMEM as described above or in medium containing 0.9 mg/l galactose. Cells (7.5 × 10^4^ to 1 × 10^5^) were seeded on day 0 of the experiment, and were counted on day 1, day 2 and day 3.

Stable inducible cell lines expressing C-terminal FLAG-tagged versions of proteins were generated as described previously (21,22) using GeneJuice (Novagen) or Lipofectamine 3000 (Invitrogen) as transfection reagents following the manufacturer's instructions.

*Gtpbp6^−^^/^^−^* cell line was generated utilizing Alt-R CRISPR–Cas9 system (Integrated DNA Technologies) according to the manufacturer instructions. Briefly, cells were co-transfected with Cas9 enzyme and crRNA–tracrRNA duplex. crRNA was designed to target the first exon of the *Gtpbp6* gene. Clones were screened for mutations in GTPBP6 gene using the Alt-R Genome Editing Detection Kit (Integrated DNA Technologies) and the presence of mutations leading to premature stop codons formation in *Gtpbp6* were confirmed by Sanger sequencing.

### Cell lysates and western blotting

Cells were lysed in nonionic lysis buffer (50 mM Tris–HCl pH 7.4, 130 mM NaCl, 2 mM MgCl_2_, 1% NP-40, 1 mM PMSF and 1× Protease Inhibitor Cocktail (Roche)). Cell lysates were separated on 10–18% Tris–Tricine gel, transferred to nitrocellulose blotting membrane AmershamTM Protran™ 0.2 μm NC (GE Healthcare) and visualized using specific antibodies.

### Northern blotting

Total RNA was isolated from whole cell extracts using TRIzol reagent (Invitrogen), loaded onto a denaturing formaldehyde/formamide 1.2% agarose gel and blotted to Amersham Hybond™-N membrane (GE Healthcare). After UV-crosslinking, the membranes were incubated with [^32^P]-radiolabelled probes targeting the specific mitochondrial RNAs and scanned with Typhoon imaging system (GE Healthcare).

### [^35^S]Methionine *de novo* synthesis

[^35^S]Methionine labeling was performed as described previously (23,24). To monitor *de novo* mitochondrial protein synthesis cells were treated with 100 μg/ml emetine (Roth) to block cytosolic translation and incubated in the presence of 0.2 mCi/ml [^35^S]Methionine for 1 h. Mitochondrial translation products were visualized with Typhoon imaging system (GE Healthcare).

### Mitochondria isolation and sucrose density gradient ultracentrifugation

Mitochondria isolation from cultured cells was performed as described (13). Briefly, cells were homogenized in trehalose buffer (300 mM trehalose, 10 mM KCl, 10 mM HEPES–KOH pH 7.4, 1 mM PMSF and 0.2% BSA) using Homogenplus Homogenizer (Schuett-Biotec, Germany). After lysis (3% sucrose, 100 mM KCl, 10 mM MgCl_2_, 20 mM HEPES–KOH, pH 7.4, 1% digitonin, 1× Protease inhibitor cocktail (Roche) and 0.08 U/μl RiboLock RNase Inhibitor) 500 μg mitochondria were separated by sucrose gradient ultracentrifugation (5–30% sucrose (w/v) in 100 mM KCl, 10 mM MgCl_2_, 20 mM HEPES/KOH, pH 7.4, 1% digitonin, 1× Protease inhibitor cocktail (Roche)) at 79 000 × g, 4°C for 15 h using SW41 Ti rotor (Beckman Coulter). Fractions (1-16) were collected with BioComp fractionator, ethanol precipitated and analyzed by western blotting or mass spectrometry.

### Quantitative mass spectrometry analyses

Precipitated proteins of three biological replicates of each condition (Gradient fraction #8 of *Gtpbp6^−^^/^^−^* and WT) were dissolved with 4 M urea in 50 mM ammonium bicarbonate pH 8.0 and diluted to a final concentration of 1 M urea and 50 mM ammonium bicarbonate. Proteins were reduced and alkylated with 5 mM TCEP and 20 mM chloroacetamide, respectively. Proteins were digested with trypsin in an enzyme-to-protein ratio of 1:50 at 37°C overnight. Peptides were acidified with trifluoroacetic acid (TFA) to a final concentration of 0.5% (v/v), desalted via MicroSpin Colums (Harvard Apparatus) following manufacturer's instructions and vacuum dried. Peptides were resuspended in 2% acetonitrile/0.05% TFA and subjected to LC–MS/MS.

Peptides were measured in technical duplicates via an Q Exactive HF mass spectrometer coupled to a Dionex UltiMate 3000 UHPLC system (both Thermo Fisher Scientific) equipped with an in house-packed C18 column. Separation of peptides was accomplished via the following gradient: mobile phase A consisted of 0.1% formic acid (FA, v/v), mobile phase B of 80% ACN/0.08% FA (v/v). The gradient started at 5% B, increasing to 10% B within 3 min, followed by a continuous increase to 46% B within 74 min, then keeping B constant at 90% for 5 min. After each gradient the column was again equilibrated to 5% B for 6 min. The flow rate was set to 300 nl/min. MS1 survey scans were acquired with a resolution of 60 000, an injection time (IT) of 50 ms and an automatic gain control (AGC) target of 1 × 10^6^. Dynamic exclusion was set to 30 s and the 30 most abundant precursor ions were considered for fragmentation. MS2 spectra were acquired with a resolution of 15 000, IT was set to 128 ms and the AGC target 1 × 105. Fragmentation was enforced by higher-energy collisional dissociation (HCD) at 30% normalized collision energy.

Raw files were analyzed by MaxQuant (v. 1.6.0.1, (25)). Default settings were applied with carbamidomethylation of cysteines as fixed and oxidation of methionines as variable modifications, FDR was set to 0.01. The label-free quantification (LFQ) algorithm was enabled. MS data were searched against all reviewed human proteins according to UniProt (26 530 entries, February 2020). Perseus (v. 1.6.0.7, (26)) was used for data analysis. Three LFQ values in at least one group (WT and/or *Gtpbp6^−^^/^^−^*) were considered for further processing. Missing values were imputed based on normal distribution of LFQ values in each group. A two-sample Student's *t*-test (S0 2, permutation-based FDR 0.01 with 250 randomizations) was applied to assess statistical significance.

### Protein localization assays

Intracellular GTPBP6 localization was determined as described (24). Briefly, isolated mitochondria or mitoplasts from GTPBP6^FLAG^ tagged cell line were treated with or without Proteinase K. Samples were separated on a gel and analyzed by western blotting. To dissect whether GTPBP6 is integrated or associated with the inner mitochondrial membrane, mitochondria isolated from GTPBP6^FLAG^ cell line were incubated in 0.1 M Na_2_CO_3_ at different pH values. Samples were centrifuged at 41,000 rpm using TLA55 rotor (Beckman Coulter) for 1h. Obtained fractions were analyzed by western blotting.

### Co-immunoprecipitation

FLAG co-immunoprecipitation of GTPBP6^FLAG^-associated protein complexes was performed as described (22). Isolated mitochondria (1 mg) were lysed (20 mM HEPES–KOH pH 7.4; 100 mM KCl; 10 mM MgCl_2_; 10% glycerol; 1 mM PMSF and 1% digitonin) prior centrifugation at 16 000 × g at 4°C for 10 min. Lysates were incubated with anti-FLAG M2 Affinity Gel (Sigma) for 1h. GTPBP6^FLAG^-bound protein complexes were eluted using FLAG peptides.

### Protein purification

*Escherichia coli* BL21 strains were transfected with plasmids (pGex-6P-1) containing N-terminal GST-tagged wild type and mutant variants of GTPBP6Δ43. Cells were grown until OD600 = 0.6, induced with 1 mM IPTG and cultured overnight at 16°C. Obtained bacterial pellets were lysed in lysis buffer (50 mM Tris–HCl, pH 7.4, 300 mM KCl, 1× protease inhibitor cocktail (Roche), 1 mM PMSF) using EmulsiFlex-C3 (Avestin). Cleared cell lysates were incubated in the presence of 600 mM KCl to reduce contamination with bacterial ribosomes and ultra-centrifuged for 1 h at 250 000 × g (Rotor Type 70Ti, Beckman Coulter). Salt concentration was diluted to 300 mM prior subjecting samples to Glutathione SepharoseTM 4B beads (GE Healthcare). After overnight binding at 4°C, beads were washed seven times with buffer A (50 mM Tris–HCl, pH 7.4, 300 mM KCl, 1 mM PMSF, 2 mM DTT) and three times with buffer B (50 mM Tris–HCl, pH 7.4, 70 mM NH_4_Cl, 30 mM KCl, 7 mM MgCl_2_) and incubated overnight in buffer C (50 mM Tris–HCl, pH 7.4, 70 mM NH_4_Cl, 30 mM KCl, 7 mM MgCl_2_, 1 mM EDTA, 1 mM DTT) with addition of 100 U of PreScission™ protease (GE Healthcare) per 2 ml of beads suspension. Next day elutions were collected and samples were concentrated with Amicon^®^ Centrifugal Filter Units Ultracel-10K (Merck Millipore) and stored in 50 mM Tris–HCl, pH 7.4, 70 mM NH_4_Cl, 30 mM KCl, 7 mM MgCl_2_, 1 mM DTT and 10% Glycerol at –80°C.

*Escherichia coli* initiation factors, EF-Tu, EF-G and RRF were purified according to published protocols (27–30).

### Preparation of ribosome complexes

70S ribosomes were purified from *E. coli* MRE600; f[^3^H]Met-tRNA^fMet^ and [^14^C]Glu-tRNA^Glu^ were produced according to the published protocols (30–32).

Initiation complexes (IC) and ternary complexes were reconstituted as in (28) with following modifications. For IC, 1.5 μM of 70S ribosomes mixed with 3 μM mRNA encoding MEKF peptide, 2.6 μM of initiation factor each (IF1, IF2, IF3), 1 mM DTT, 1 mM GTP and 4.5 μM of f[^3^H]Met-tRNA^fMet^ were incubated at 37°C for 5 min. For ternary complex, 18 μM of EF-Tu was combined with 1 mM GTP, 1 mM DTT, 3 mM phosphoenol pyruvate, 0.1 mg/ml pyruvate kinase, and after 15 min of incubation at 37°C [^14^C]Glu-tRNA^Glu^ was added. Translation was performed at 37°C for 5 min after mixing 0.75 μM IC with 9 μM of ternary complex and 0.2 μM EF-G. Resulting pre-hydrolysis complexes were purified through 1.1 M sucrose cushion for 2 h at 201 000 × g. Translation efficiency was estimated by nitrocellulose filtration followed by scintillation counting of ribosome-bound tRNAs (28).

Post-hydrolysis complexes were produced when incubating pre-hydrolysis complexes with 0.1 mM puromycin at 37°C for 10 min. Efficiency of f[^3^H]Met-[^14^C]Glu-puromycin formation was estimated by reverse-phase high performance liquid chromatography using Cromolith RP-8e 100–4.6 mm column (Merck) and quantified with scintillation counting.

### Rapid kinetic measurements

The dissociation of 70S to subunits was monitored using Rayleigh light scattering at 325 nm using stopped flow apparatus (Applied Photophysics) (18,19,33). Experiments were performed in buffer A (50 mM Tris–HCl pH 7.5; 70 mM NH_4_Cl; 30 mM KCl and 7 mM MgCl_2_) at 37°C. 70S ribosomes or pre- and post-hydrolysis complexes (0.05 μM) were rapidly mixed with 1 μM of GTPBP6 (if not indicated otherwise) in the presence of 0.5 mM GTP. In control reactions, buffer A or EF-G (2 μM) and RRF (2.5 μM) were used instead of GTPBP6. To assess the nucleotide dependence of GTPBP6 induced 70S recycling, GTP was replaced with either GDP, GTPγS, ADP or ATP (each 0.5 mM) or buffer A. Phosphoenol pyruvate (3 mM) and pyruvate kinase (0.1 μg/μl) were used in experiments to determine the *K*_d_ of 70S and GTPBP6. All concentrations are indicated as the final concentrations after mixing. The reaction was recorded for at least 75 s and data fitting was performed on normalized curves obtained by averaging of 5–8 traces with GraphPad Prism 5.0 for Windows (GraphPad Software, www.graphpad.com) or with TableCurve 2D (Systat Software).

### Quantification and statistical analysis

The protein and RNA levels were measured from western blots and northern blots using Typhoon imaging system (GE Healthcare) and quantified with ImageQuant TL software (GE Healthcare). The results from 3 independent experiments are presented as percentages relative to wild type control ± SEM (see figure legends for details).

## RESULTS

### GTPBP6, a homolog of bacterial HflX, is a mitochondrial matrix protein

**Figure 1.**
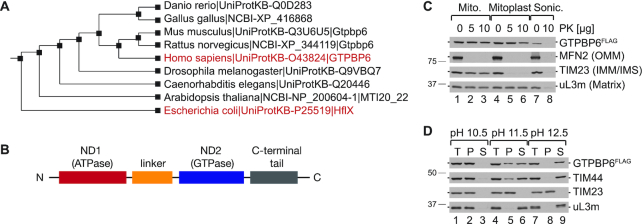
GTPBP6 is a mitochondrial protein homologous to bacterial HflX. (**A**) Cluster analysis of human GTPBP6 and bacterial HflX using P-POD (Princeton Protein Orthology Database (http://ppod.princeton.edu/)). (**B**) Domain arrangement of HflX and GTPBP6. Nucleotide binding domains (ND1 and ND2) are indicated in red and blue, respectively, the α-helical linker in orange and the C-terminal tail in grey. (**C**) Human GTPBP6 is a mitochondrial matrix protein. Mitochondria were isolated from HEK293T cells expressing C-terminal FLAG tagged GTPBP6. Intact mitochondria (lane 1–3), mitoplasts (lane 4–6) and sonicated mitochondria (lane 7–8) were treated with Proteinase K (PK) as indicated. Samples were analyzed by western blotting using antibodies against MFN2, TIM23 and uL3m as markers for the outer mitochondrial membrane (OMM), the inner mitochondrial membrane (IMM), the intermembrane space (IMS), and the matrix, respectively. GTPBP6^FLAG^ was detected using antibodies against the FLAG epitope. (**D**) GTPBP6 is peripherally associated with the inner mitochondrial membrane. Mitochondrial membrane proteins were extracted using sodium carbonate solutions with different pH. Samples (total, T; pellet, P; and supernatant, S) were analyzed by western blotting using antibodies as indicated.

Cluster analysis of human GTPBP6 reveals that this GTPase is highly conserved and homologous to the bacterial ribosome-recycling factor HflX (Figure 1A). *E. coli* HflX contains two nucleotide-binding domains, an N-terminal putative ATP-dependent RNA helicase domain (ND1) and a C-terminal GTPase domain (ND2) connected by an α-helical linker (20) (Figure 1B). Human GTPBP6 and *E. coli* HflX share ∼30% sequence identity and have a similar domain arrangement (Supplementary Figure S1). Human MitoCarta2.0 database and MitoProt II (v1.101) suggest that GTPBP6 is a mitochondrial protein. To validate the mitochondrial localization, we tested the sensitivity of GTPBP6 to Proteinase K digestion in isolated mitochondria and mitoplasts. As antibodies against the endogenous GTPBP6 are unavailable, we ectopically expressed a C-terminal FLAG-tagged version of GTPBP6 and tested the protein localization in mitochondria isolated from these cells. GTPBP6^FLAG^ appears to be inside mitochondria as it is protected against Proteinase K treatment even when the outer membrane has been removed, similar to the matrix marker uL3m (Figure 1C). Sodium carbonate extraction experiments with isolated mitochondria show that some GTPBP6^FLAG^ is still present in the membrane fraction at pH 11.5, but it is completely extracted at higher pH, similar to TIM44, a protein of the import motor peripherally associated to the inner mitochondrial membrane (Figure 1D) (34,35). These data suggest that GTPBP6 is a mitochondrial matrix protein peripherally associated to the inner mitochondrial membrane like other mitochondrial GTPases such as GTPBP5 or GTPBP10 (13,16).

### Excess GTPBP6 inhibits mitochondrial translation by promoting ribosome dissociation

**Figure 2.**
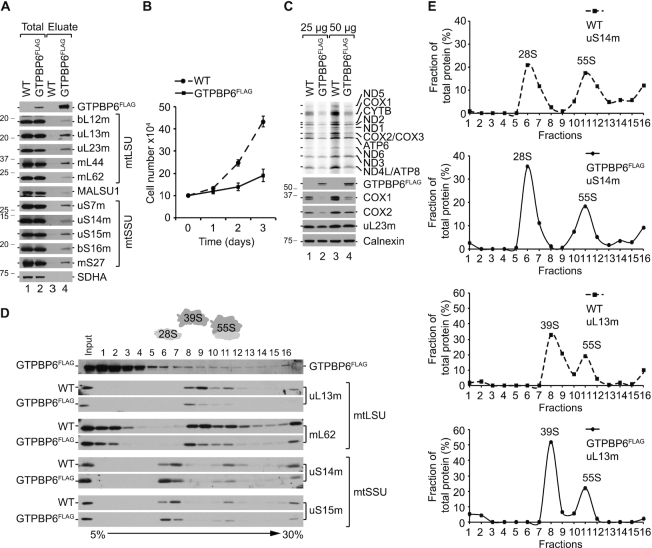
Elevated levels of GTPBP6 affect mitochondrial translation. (**A**) GTPBP6 interacts with the mitochondrial ribosome. Lysed mitochondria (1 mg) isolated from HEK293T-GTPBP6^FLAG^ and -wild type (WT) cells were subjected to FLAG-immunoprecipitation. Complexes were eluted using FLAG peptide. Samples (3% total; 100% eluate) were analyzed by western blotting using specific antibodies as indicated. SDHA (Subunit A of the succinate dehydrogenase) was used as a negative control. (**B**) GTPBP6 overexpression affects cell growth. HEK293T-GTPBP6^FLAG^ and wild type (WT) cells were cultured in galactose-containing media in the presence of 500 ng/ml tetracycline. Cells were counted after 1d, 2d and 3d. (*n* = 3; mean ± SD). (**C**) Elevated GTPBP6 protein levels reduce mtDNA expression. Synthesis of mtDNA-encoded proteins in HEK293T-GTPBP6^FLAG^ and wild type (WT) cells was analyzed by [^35^S]Methionine *de novo* incorporation. Samples were subjected to SDS-PAGE followed by autoradiography and western blot analyses. Calnexin was used as loading control. (**D**) Overexpression of GTPBP6 results in accumulation of mtSSU and mtLSU. Ribosome profiles were analyzed by sucrose density gradient centrifugation using lysed mitochondria (0.5 mg) isolated from HEK293T-GTPBP6^FLAG^ and wild type (WT) cells. Fractions (1-16) were analyzed by western blotting using antibodies as indicated (Input, 10% of material applied on a sucrose gradient). (**E**) Quantification of the relative distribution of mtSSU, mtLSU and 55S ribosomes using antibodies against uS14m and uL13m, respectively (100% = total protein in all fractions of tested cell line).

Bacterial HflX preferentially associates with the large ribosomal subunit (LSU) (18,36,37). By analogy, we tested the interaction of GTPBP6 with the mitochondrial ribosome by performing FLAG immunoprecipitation. GTPBP6^FLAG^ used as a bait co-precipitates all tested ribosomal proteins suggesting that GTPBP6 binds either to both ribosomal subunits or to the assembled 55S complex (Figure 2A). Induction of GTPBP6^FLAG^ expression results in a slower cell growth and rapid media acidification. This phenotype is even more pronounced in galactose-containing media that was used to force mitochondria to produce ATP via OXPHOS (Figure 2B), resulting in a significant (by 2.9-fold) reduction of growth rate, suggesting that GTPBP6 overexpression negatively affects mitochondrial function. To test whether GTPBP6 overexpression has a direct effect on mitochondrial translation, we performed [^35^S]Methionine ([^35^S]Met) labeling of *de novo*-synthesized mtDNA-encoded proteins. Synthesis of mtDNA-encoded proteins is markedly decreased upon overexpression of GTPBP6^FLAG^ compared to wild type, resulting in reduced protein steady state levels as shown for COX1 and COX2 (Figure 2C).

HflX acts as a ribosome-recycling factor (18,19,38–40). To test whether also GTPBP6 might facilitate ribosome dissociation into subunits, we performed sucrose density gradient centrifugation to analyze the ratio of mtLSU and mtSSU relative to 55S ribosomes (Figure 2D, Supplementary Figure S2). Indeed, the fraction of free mtLSU and mtSSU increases upon overexpression of GTPBP6^FLAG^ at the cost of 55S ribosomes (Figure 2D, E). Quantification of the ribosome profiles yields the ratio of mtSSU:mtLSU:55S of 1.2:1.7:1 in wild type cells, which increases to 2.7:3.3:1 upon GTPBP6 overexpression (Figure 2E), suggesting that mtSSU and mtLSU accumulate in GTPBP6^FLAG^ expressing cells. Thus, the decrease of mitochondrial gene expression can be explained by the reduced number of translationally active 55S mitochondrial ribosomes caused by the ribosome-recycling activity of GTPBP6.

### GTPBP6 dissociates 70S ribosomes *in vitro*

We next validated the ribosome-recycling activity of GTPBP6 *in vitro*. As a reconstituted *in vitro* translation system with 55S ribosomes is not available, we tested the ribosome dissociation activity of purified recombinant GTPBP6 using isolated 70S ribosomes from *E. coli*. Such hybrid systems have been successfully utilized to investigate the functions of human mitochondrial translation factors such as mtIF2, mtEFTu, mtEFG2, mtRF1a or ICT1 (41–44).

**Figure 3.**
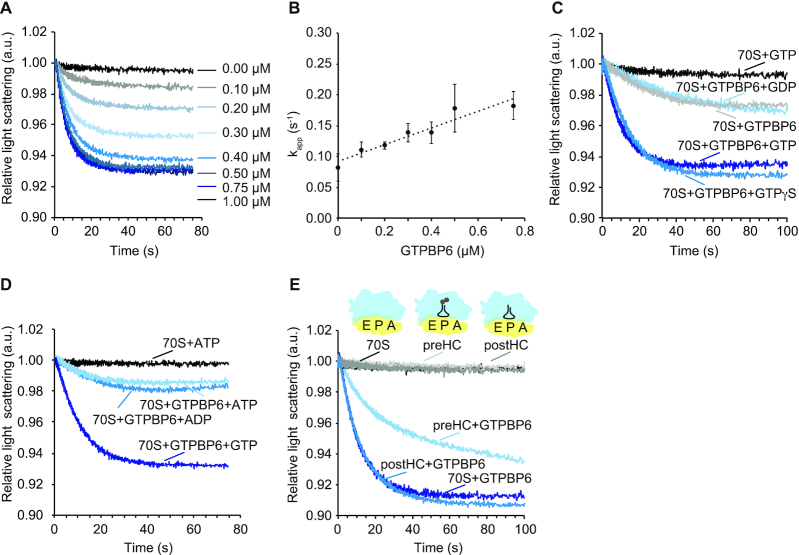
GTPBP6 splits 70S ribosomes *in vitro*. (**A**) Change in light scattering as a response to GTPBP6-induced dissociation of 70S ribosomes into subunits. 70S ribosomes (0.05 μM) were rapidly mixed with indicated concentrations of GTPBP6 in the presence of 0.5 mM GTP. The scattered light intensity at 325 nm was measured in a stopped-flow apparatus. Each curve represents the average of 5–8 individual traces. (**B**) GTPBP6 concentration dependence of 70S ribosome recycling. The apparent rate constants (*k*_app_) obtained by fitting the light scattering curves on Figure 3A with one-exponential function, were plotted against GTPBP6 concentration. As *k*_app_ showed linear dependence of GTPBP6 concentration, linear fitting provided dissociation and association constants (intercept and slope), ratio of which represents *K*_d_ of reaction. (**C**) Dissociation of 70S ribosomes by GTPBP6 depends on GTP-binding but not on GTP-hydrolysis. 70S ribosomes (0.05 μM) were mixed rapidly with GTPBP6 (1 μM) in the absence or presence of GDP, GTP or GTPγS (0.5 mM each). *k*_app_, was estimated to be 0.09 ± 0.01 s^−1^ in the presence of GTP and 0.08 ± 0.01 s^−1^ in the presence of GTPγS. (**D**) GTPBP6 does not require ATP for 70S dissociation. Experiments were performed as in (A) and (C) in the presence of GTP, ATP or ADP (0.5 mM each). (**E**) GTPBP6 splits post-hydrolysis complexes. Dissociation of vacant 70S *E. coli* ribosomes (70S), pre-hydrolysis complexes (preHC) and post-hydrolysis complexes (postHC) (0.05 μM each) by GTPBP6 (1 μM) was determined in the presence of GTP (0.5 mM).

Ribosome dissociation to subunits can be monitored as a change in the intensity of scattered light (18,19,45). To monitor the dissociation in real time, we rapidly mixed 70S ribosomes with increasing concentrations of GTPBP6 in the presence of excess GTP in a stopped-flow apparatus and recorded the change in light scattering (Figure 3A). GTPBP6 facilitated ribosome dissociation in a concentration dependent manner. The apparent rate constants (*k*_app_) of reactions were plotted as a function of GTPBP6 concentration (Figure 3B), and the slope of the linear fit provided the association rate constant of about 0.13 μM^−1^s^−1^. The dissociation rate constant of the 70S-GTPBP6 complex, determined from the Y-axis intercept, is about 0.09 s^−1^. The *K*_d_ of 0.7 μM, which was calculated from both rate constants, is comparable to the *K*_d_ value of 1.0–1.5 μM that was reported for the HflX-70S complex (18).

The dissociation of 70S by GTPBP6 was independent of GTP hydrolysis (Figure 3C), as the reaction with non-hydrolysable GTP analog GTPγS was as efficient as with GTP. In contrast, the reaction with GDP-bound or apo-GTPBP6 was very slow (Figure 3C), suggesting that the ribosome recycling activity of GTPBP6 depends on GTP binding, but not on GTP hydrolysis. As GTPBP6 contains a putative ATP-dependent RNA-helicase domain (46), we also tested whether ATP affects GTPBP6-facilitated ribosome dissociation and found that neither ATP nor ADP can replace GTP (Figure 3D).

To analyze GTPBP6 action on functional ribosome complexes, we utilized 70S ribosomes programmed with mRNA and carrying fMet-Glu-tRNA^Glu^ in the P site (pre-hydrolysis complex, preHC) or 70S ribosomes with deacylated tRNA^Glu^ in the P site (post-hydrolysis complex, postHC); both complexes had an empty A site. Similarly to bacterial recycling factor RRF together with EF-G (Supplementary Figure S3B), GTPBP6 facilitated rapid dissociation of postHC or vacant 70S ribosomes, but was less efficient on ribosomes with a peptidyl-tRNA in the P site (Figure 3E, Supplementary Table S2), suggesting that GTPBP6 activity is inhibited on translationally active ribosomes. These data show that GTPBP6 indeed acts as a ribosome-recycling factor facilitating the dissociation of vacant ribosomes as well as postHC into subunits in a GTP-dependent manner.

### GTPBP6 loss stalls mtLSU biogenesis

**Figure 4.**
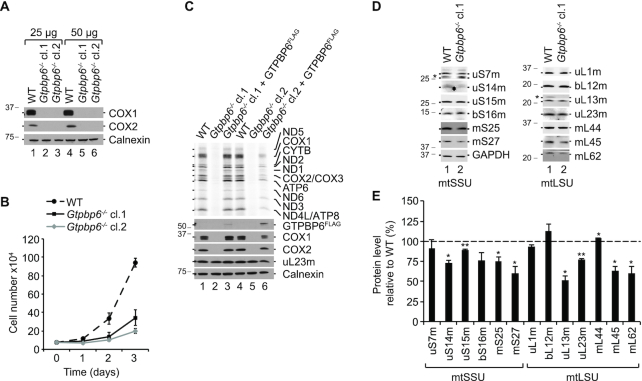
GTPBP6 is essential for mitochondrial gene expression. (**A**) Loss of GTPBP6 inhibits synthesis of mtDNA-encoded proteins. Cell lysates from HEK293T-wild type (WT) and *Gtpbp6^−^^/^^−^* cells were analyzed by western blotting using antibodies as indicated. Two clones obtained from CRISPR/Cas9 application followed by FACS were used, indicated as cl.1 and cl.2. (**B**) Ablation of GTPBP6 affects cell growth. HEK293T-wild type (WT) and *Gtpbp6^−^^/^^−^* cells were seeded into 6-well plates using DMEM media and counted after 1 d, 2 d and 3 d (*n* = 3; mean ± SD). The growth rate of *Gtpbp6^−^^/^^−^* clone 1 and clone 2 was estimated to be reduced by 1.8- and 2.5-fold, respectively compared to WT. (**C**) Essential role of GTPBP6 in translation of mtDNA-encoded proteins. HEK293T-wild type (WT), *Gtpbp6^−^^/^^−^* and *Gtpbp6^−^^/^^−^* cells inducibly expressing GTPBP6^FLAG^ were analyzed by [^35^S]Methionine *de novo* incorporation. Samples were analyzed by autoradiography and western blotting, respectively. (**D**) The effect of GTPBP6 ablation on synthesis of mitoribosomal proteins. Cell lysates from HEK293T-wild type (WT) and *Gtpbp6^−^^/^^−^* cells (cl. 1) were analyzed by western blotting using Typhoon imaging system (GE Healthcare). (*) indicates unspecific signal. GAPDH was used as loading control. (**E**) Relative protein levels were quantified from (D) using ImageQuant TL software. The expression level of tested proteins in wild type cells are marked as dashed line (100%) (*n* = 3; mean ± SEM; * *P* < 0.05; ** *P* < 0.01).

To test whether GTPBP6 is essential in mitochondria, we generated knockout cell lines applying CRISPR/Cas9 technology. For detailed analysis, we have chosen two clones, both containing mutations in exon 1 leading to premature stop codons (Supplementary Figure S4A–C). Analysis of steady-state protein levels in the deletion clones shows a complete lack of mtDNA-encoded proteins COX1 and COX2, suggesting that GTPBP6 is required for mitochondrial gene expression (Figure 4A). Ablation of GTPBP6 inhibits cell growth by ∼2-fold compared to wild type (Figure 4B) and generally abolishes mtDNA expression as monitored by [^35^S]Met *de novo* incorporation *in vivo* (Figure 4C).

To validate that the loss of GTPBP6, rather than an off-target effect, leads to the inhibition of mitochondrial translation, we generated rescue cell lines by integrating FLAG-tagged GTPBP6 into the Flp-In cassette of the HEK293T *Gtpbp6^−^^/^^−^* cell lines allowing inducible expression of GTPBP6^FLAG^. Because overexpression of GTPBP6 has a negative effect on mitochondrial translation (Figure 2C), we titrated the inducer to a minimum to avoid false-negative results (Supplementary Figure S4D, E). Indeed, low-level expression of GTPBP6^FLAG^ restored the phenotype in *Gtpbp6^−^^/^^−^* clone 1 confirming the specificity of the knockout and the functionality of the FLAG tagged variant of GTPBP6 (Figure 4C). In clone 2, mitochondrial translation was only partially restored possibly because the expression of GTPBP6^FLAG^ was somewhat higher, which could have an inhibitory effect on translation.

**Figure 5.**
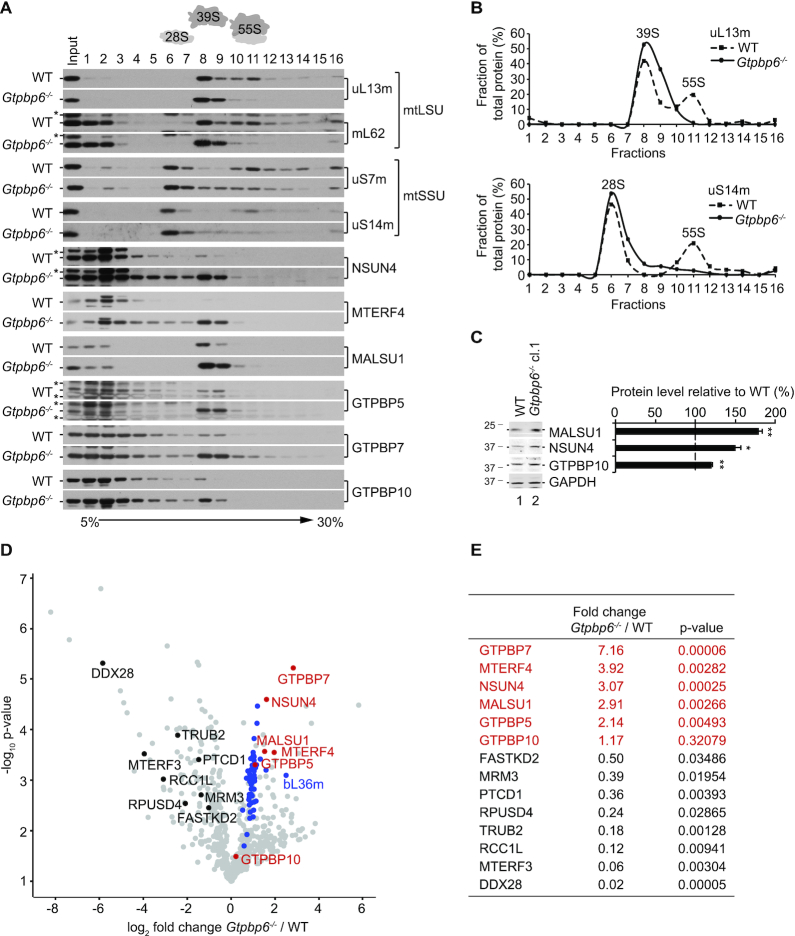
GTPBP6 is required for 55S ribosome assembly. (**A**) GTPBP6 ablation results in accumulation of mtSSU and mtLSU. Mitochondria (0.5 mg) were isolated from HEK293T-wild type (WT) and *Gtpbp6^−^^/^^−^* cells (cl. 1). Ribosomes and ribosomal subunits were separated by sucrose density gradient centrifugation. Fractions (1-16) were analyzed by western blotting using antibodies as indicated. (*) indicates unspecific signal. (**B**) Distribution of mtSSU, mtLSU and 55S ribosomes calculated as percentage of uS14m and uL13m in each fraction. (**C**) GTPBP6 loss affects mitochondrial ribosome assembly factors. Cell lysates isolated from HEK293T-wild type (WT) and *Gtpbp6^−^^/^^−^* cells (cl. 1) were analyzed as described in Figure 4E (*n* = 3; mean ± SEM; * *P* < 0.05; ** *P* < 0.01). (**D, E**) mtLSU composition in *Gtpbp6^−^^/^^−^* cells. Proteins isolated from fraction 8 corresponding to mtLSU were analyzed by label-free quantitative mass spectrometry (*Gtpbp6^−^^/^^−^* versus WT). Ribosomal proteins of mtLSU are labeled in blue, assembly factors accumulating in *Gtpbp6^−^^/^^−^* are indicated in red and factors, which are reduced upon GTPBP6 ablation are marked in black (*n* = 3).

To test whether mtDNA expression deficiency is due to defects in mitochondrial translation, transcripton or mt-RNA stability, we quantified the steady-state levels of the mitochondrial rRNAs, *MTRNR1* (12S rRNA) and *MTRNR2* (16S rRNA), and of mitochondrial mRNAs, *MTCO1* (COX1 mRNA) and *MTCO2* (COX2 mRNA) by Northern blotting (Supplementary Figure S4F, G). We did not observe significant changes in the steady-state levels of tested mt-RNAs suggesting that the defects in mtDNA expression are specific for mitochondrial translation. We also did not detect severe changes in the steady state levels of ribosomal proteins in clone 1 (Figure 4D, E). However, when we tested whether these proteins assemble into a functional mitochondrial ribosome, we found a significant reduction in the fraction of 55S ribosomes and a considerable accumulation of 28S mtSSU and 39S mtLSU, which can explain the defects in mitochondrial translation (Figure 5A, B). Notably, we observed a strong enrichment of NSUN4, MTERF4, MALSU1 and the GTPases GTPBP5, GTPBP7 and GTPBP10 in the 39S mtLSU fractions, which suggests accumulation of mtLSU assembly intermediate(s) at a late stage of maturation (12,13,16,47,48). The steady-state protein levels of NSUN4, MALSU1 and GTPBP10 were significantly increased in *Gtpbp6^−^^/^^−^* cells compared to wild type (Figure 5C). To determine the composition of the complexes accumulating in fraction 8 (Figure 5A) we applied a label-free mass spectrometry approach. Interestingly, all 52 ribosomal proteins of the mtLSU were detected in WT and *Gtpbp6^−^^/^^−^* samples and in most cases were even enriched in the absence of GTPBP6 (Figure 5D, Supplementary Table S3). This also includes bL36m, one of the last assembled ribosomal proteins (16) suggesting that the mtLSU in *Gtpbp6^−^^/^^−^* cells represents an almost matured mtLSU with a complete set of ribosomal proteins. However, the accumulation of numerous assembly factors on the mtLSU complex(es) (Figure 5A, D, E), which in this state is incompetent in 55S ribosome formation, suggests that final folding or rearrangements of ribosomal proteins or/and rRNA might not be completed in the absence of GTPBP6. Thus, GTPBP6 ablation leads to the accumulation of late mtLSU assembly intermediate(s) containing NSUN4-MTERF4, MALSU1, GTPBP5, GTPBP7 and GTPBP10 resulting in a drastic loss of mature 55S ribosomes and inhibition of mitochondrial translation.

### Ribosome recycling and biogenesis are distinct GTPBP6 functions

As GTPBP6 plays a dual role in mitochondrial translation by facilitating ribosome recycling and biogenesis, we asked whether these activities locate to particular regions of GTPBP6. Based on the sequence similarities to HflX, we identified residues for mutational analysis (Supplementary Figure S1). GTPBP6 is expected to contain two nucleotide-binding domains, the putative ATPase domain (ND1) and the GTPase domain (ND2) (46). Although the experimental evidence for the functional roles of specific residues in ND1 of HflX is lacking, Arg90 and Asp102 in ND1 were predicted to be involved in ATP binding (46). These residues correspond to Lys187 and Asp199 in human GTPBP6 (Supplementary Figure S1). We changed these charged amino acids to alanine resulting in two ND1 domain mutations, K187A and D199A. In the GTPase domain, Gly235 in HflX of *S. solfataricus* is essential for GTP hydrolysis, as its mutation to proline leads to a complete loss in the GTPase activity (49). This residue is conserved and corresponds to Gly352 in human GTPBP6. Comparison of GTPBP6 to human GTPBP10, another member of the OBG-HflX-like GTPase superfamily, suggested yet another potentially important residue in the G5 motif, Ser437, which corresponds to Ser325 in the GTPase domain of GTPBP10. Mutation of Ser325 to proline results in impaired ribosome association (13). This conserved residue has been predicted to abolish GTPase activity in bacterial ObgE (50).

**Figure 6.**
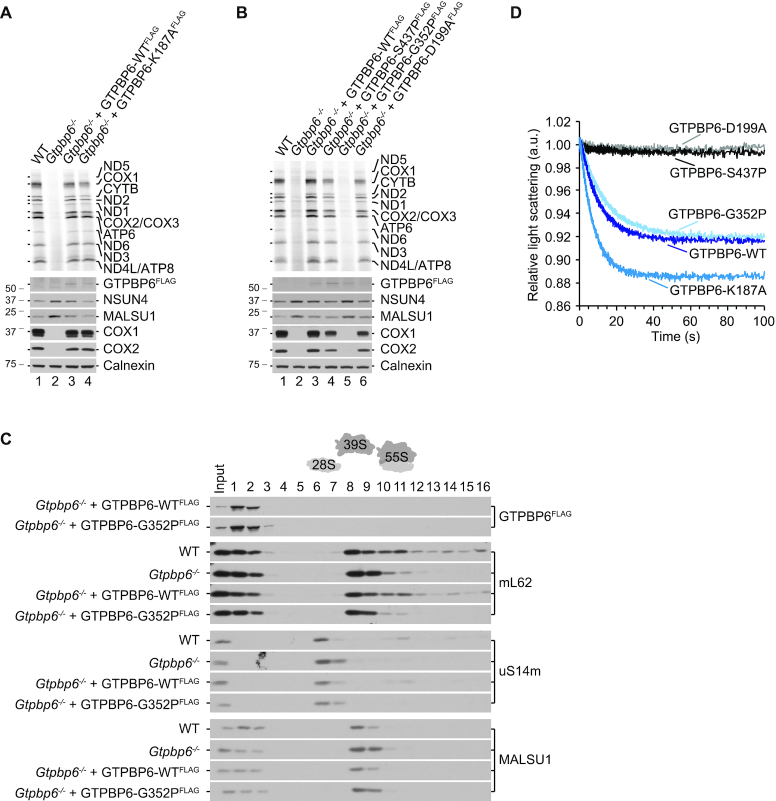
Mutation analyses of GTPBP6. (**A**, **B**) Residue G352 is required for GTPBP6 acting as a ribosome biogenesis factor. *Gtpbp6^−^^/^^−^* cells expressing wild type or mutant variants of GTPBP6 were analyzed by [^35^S]Methionine *de novo* incorporation. GTPBP6^FLAG^ protein expression was induced with tetracycline (GTPBP6-WT: 1 ng/ml; -K187A: 1 ng/ml; -D199A: 12 ng/ml; -G352P: 10 ng/ml; -S437P: 25 ng/ml) and adjusted to the level comparable to the one in Figure 4C, lane 3. All mutants were generated in *Gtpbp6^−^^/^^−^* cl. 1. Samples were analyzed by autoradiography and western blotting. (**C**) GTPBP6-G352P mutant is incompetent in forming functional 55S ribosomes. Mitochondrial lysates (0.5 mg) from WT, *Gtpbp6^−^^/^^−^* (cl. 1), *Gtpbp6^−^^/^^−^*+GTPBP6-WT^FLAG^ and *Gtpbp6^−^^/^^−^*+GTPBP6-G352P^FLAG^ were analyzed by sucrose density gradient centrifugation as in Figure 5A. (**D**) GTPBP6 mutants G352P and K187A facilitate dissociation of 70S ribosomes into subunits. Purified mutant and the wild type proteins (1 μM) were mixed with 70S ribosomes (0.05 μM) in the presence of GTP (0.5 mM GTP) as described in Figure 3, and the dissociation was monitored by a change in light scattering.

First, we performed rescue experiments by transfecting *Gtpbp6^−^^/^^−^* cells cl. 1 with pcDNA5/FRT/TO encoding GTPBP6^FLAG^ with the respective mutations. The expression levels of mutant proteins were adjusted to a level comparable to the wild type GTPBP6^FLAG^ (Figure 4C, lane 3). We tested mutant cell lines for *de novo* synthesis of mtDNA-encoded proteins by [^35^S]Met incorporation (Figure 6A, B). GTPBP6 with a K187A mutation was as active in alleviating the inhibitory effect of GTPBP6 deletion as wild type protein. The D199A and S437P mutants restored mitochondrial translation, albeit not to the same extent as wild type GTPBP6^FLAG^. The G352P mutant did not rescue the *Gtpbp6^−^^/^^−^* phenotype. Interestingly, assembly factors NSUN4 and MALSU1 accumulated in G352P mutant similarly as in *Gtpbp6^−^^/^^−^* cells suggesting that maturation of the mtLSU is blocked (Figure 6A, B). Accordingly, the profile of mitochondrial ribosome assembly revealed the accumulation of mtLSU intermediates that are incompetent in 55S ribosome formation in G352P mutants comparable to *Gtpbp6^−^^/^^−^* cells (Figure 6C), suggesting the cause for the overall translation defect.

Next, we tested these mutant variants of GTPBP6 in their activity to facilitate 70S ribosome dissociation *in vitro*. In contrast to the wild type GTPBP6, mutant variants D199A and S437P lost the ability to split vacant 70S ribosomes. However, mutant G352P facilitated ribosome dissociation as efficient as wild type GTPBP6. Mutant K187A was even more active than the wild type GTPBP6 (Figure 6D). The results with the D199A and S437P mutants suggest that the loss of *in vitro* ribosome-recycling activity of GTPBP6 is not detrimental for mitochondrial translation. *Vice versa*, the intact recycling activity of the G352P mutant is not sufficient to restore mitochondrial translation. Thus, it is likely that the two activities of GTPBP6, as a ribosome biogenesis factor and as ribosome recycling factor, are physically separated and require different regions of the factor.

## DISCUSSION

We show that GTPBP6, a so far uncharacterized human protein, localizes to mitochondria where it plays a role in ribosome assembly, in addition to facilitating ribosome recycling. Unlike its bacterial homolog HflX, which is non-essential at normal growth conditions, GTPBP6 is required for mitochondrial gene expression and cell survival. Its exact concentration in the cell is crucial as also elevated GTPBP6 levels in human cells are harmful for mitochondrial translation and cell growth due to depletion of the translating ribosomes pool and accumulation of mtSSU and mtLSU.

Our findings establish that the ribosome-recycling activity of GTPBP6/HflX is a conserved feature across different domains of life. Experiments in heterologous *in vitro* system demonstrate that GTPBP6 promotes rapid dissociation of *E. coli* vacant ribosomes and ribosomes with a deacylated tRNA at the P site like canonical ribosome recycling factor RRF that operates together with EF-G. Also human mitochondria employ mtRRF and mtEFG2 (42,51) and it is unclear whether GTPBP6 fulfills a complementary and/or specific role in recycling specific ribosomal complexes. HflX has been proposed to rescue ribosomes arrested at stress conditions (18,39,40). Stalled ribosomal complexes are formed if the mRNA is degraded from the 3′ end, aminoacyl tRNAs are depleted, during certain antibiotic treatments or under heat shock conditions. Neither GTPBP6 (this study) nor HflX (18) split the 70S ribosomes with a peptidyl-tRNA in the P site (preHC) as efficiently as ribosomes with a deacylated tRNA in the P site (postHC). It was proposed that the preceding action of peptidyl-tRNA hydrolases like ArfB or ArfA-RF2 might be required before HflX is able to recycle ribosomes (18). Also human mitochondria contain peptidyl-tRNA hydrolases such as C12ORF65 and ArfB homolog ICT1 (mL62) which may act on translationally arrested ribosomes (52–54). C12ORF65 and ICT1 belong to the same protein family as bacterial termination factors 1 and 2 that catalyze peptidyl-tRNA hydrolysis during termination of translation. However, C12ORF65 and ICT1 lack motifs for stop codon-specific translation termination activity, which prompted the suggestion that they might be involved in ribosome rescue in mitochondria (44,55–57). Although ICT1 has been identified as a ribosomal protein located in the central protuberance of the mtLSU (44,58,59), it has also been shown to exhibit codon-independent, but ribosome-dependent peptidyl release activity *in vitro* (44). It is possible that a free pool of ICT1 is available to terminate arrested ribosomes (60) that could be then recycled by GTPBP6. HflX has been shown to recycle ribosomes in different bacterial species (18,19,38–40). The functional importance and regulation of HflX is different depending on the organism. In *E. coli* and *S. aureus* expression of HflX is upregulated during heat stress (18,38). In *Listeria monocytogenes* macrolides and lincosamides promote ribosome stalling which in turn induces the expression of HflX (39). In contrast, human GTPBP6 is probably expressed constitutively in all tissues (46) and its expression must be tightly controlled, as abnormally high GTPBP6 levels in lymphoblasts have been associated with language impairment in men with Klinefelter's syndrome (61).

In contrast to the 70S splitting activity of *E. coli* RRF, which depends on GTP hydrolysis by EF-G (Supplementary Figure S3A) (28), GTPBP6 requires only GTP binding but not hydrolysis to dissociate the 70S ribosomes. It is possible that GTP hydrolysis induces the release of GTPBP6 from the LSU, as suggested for HflX (18). This indicates that GTPBP6 could act as an anti-association factor before GTP is hydrolyzed. Nonetheless, the rapid kinetics of 70S ribosome dissociation by GTPBP6 is typical for a recycling factor and differs from the slow reaction caused by bacterial anti-association factor IF3, which binds to already dissociated subunits and prevents their re-association (Supplementary Figure S3C) (28). It is reasonable to assume that GTPBP6 can act both as a recycling and as an anti-association factor.

Although the binding site of GTPBP6 on the bacterial or the mitochondrial ribosome is yet to be determined, it is feasible to assume that as a translational GTPase with conserved G-motifs GTPBP6 binds to the GTPase associated centre of the ribosome. The ribosome splitting function indicates that GTPBP6 might also reside at the interface of the two subunits. Thus, GTPBP6 probably binds on the 50S subunit to the similar location as HflX or a canonical ribosome recycling factor such as mtRRF (18,62). Superimposed *E. coli* HflX-bound 50S and human 39S mtLSU demonstrate structural conservation along the HflX binding site that encompasses A- and P-loops at the PTC, helix 89, which connects the PTC to the sarcin-ricin loop (SRL), and SRL (Supplementary Figure S5). Thus, it is conceivable that GTPBP6 might act on a similar position on the bacterial 50S as well as on the 39S mtLSU.

Most importantly, we demonstrate that human GTPBP6 has acquired an additional role as a ribosome biogenesis factor. Although it has been proposed that also HflX might play a role in ribosome assembly in *E. coli* (37), it is unlikely that HflX is a *bona fide* ribosome biogenesis factor as HflX loss does not show any assembly defect under laboratory growth conditions and its expression is induced by heat shock (18). In contrast, ablation of GTPBP6 leads to stalled mitochondrial ribosome biogenesis although all 52 ribosomal proteins of the mtLSU are assembled. GTPBP6 loss is characterized by an accumulation of mtLSU complex(es), enriched in NSUN4-MTERF4, MALSU1 and the GTPases GTPBP5, GTPBP7 and GTPBP10 (Figure 5A, D, E). These factors are required for mtLSU assembly at late maturation stages and thus for 55S ribosome formation (12–14,16,47,48,63,64). A similar assembly intermediate also accumulates upon loss of GTPBP5, which is required for 16S modification within the peptidyl transferase center (PTC) catalyzed by MRM2 (16,17). It has been proposed that GTPBP5 is part of a quality control system monitoring the maturation of the PTC to prevent premature subunit joining (16). It is tempting to speculate that GTPBP6 might also act as an anti-association factor to ensure late maturation steps of the mtLSU. However, in contrast to GTPBP5 loss, GTPBP6 deficiency results in the accumulation of mtLSU complex(es) containing bL36m suggesting that GTPBP6 acts downstream of GTPBP5, GTPBP7 and GTPBP10. Although we showed that only GTP-bound GTPBP6 dissociates the ribosomes efficiently, it is tempting to assume that GTP is also required for the late maturation steps of the ribosome. As proposed for other mitochondrial GTPases (12,16), GTPBP6 release from the mtLSU, likely coupled to GTP hydrolysis, might facilitate mtSSU joining.

It is reasonable to assume that the ribosome recycling and biogenesis activities of GTPBP6 are independent of each other and employ different structural elements of the factor. Ribosome recycling is likely catalyzed by the conserved GTPase ND2 domain of GTPBP6/HflX (65), whereas the variable ATP binding domain ND1 might have acquired different specialized functions in various organisms (46). In *E. coli* ND1 appears to act as an ATP-dependent RNA helicase rescuing heat-damaged ribosomes, whereas the archaeon *S. solfataricus* lacks the domain altogether (20,66). Our data indicate that the mutation in the ND2 domain which impairs GTP hydrolysis in *S. solfataricus* HflX (49), does not impair GTPBP6-dependent recycling of 70S ribosomes *in vitro*, but fails to rescue the mitochondrial ribosome assembly deficiency. If the G352 residue is indeed important for the GTPase activity of GTPBP6, this finding suggests that GTP hydrolysis is required either for the specific function of GTPBP6 in ribosome biogenesis, or for GTPBP6 dissociation from the ribosome. Contrary to the G352P mutation, D199A and S437P replacements abolish the activity of GTPBP6 in ribosome recycling, however, they are able to restore the mitochondrial translation phenotype in *Gtpbp6^−^^/^^−^* cells. Thus, ribosome-recycling activity might not be an essential function of GTPBP6, possibly because it can be covered by mtRRF-mtEFG2. However, it might be crucial under certain stress conditions like in bacteria or when the canonical recycling machine mtRRF-EFG2 is non-functional. In summary, our findings demonstrate that GTPBP6 is a versatile protein with a dual function in human mitochondria and suggest that its role in ribosome biogenesis is essential for mitochondrial translation.

## Supplementary Material

gkaa1132_Supplemental_Files
