# Supplementary material for: Dual function of GTPBP6 in biogenesis and recycling of human mitochondrial ribosomes
Source: Nucleic Acids Res. 2020 Dec 2;48(22):12929–42. doi: 10.1093/nar/gkaa1132 (PMC7736812; doi:10.1093/nar/gkaa1132)
Supplement: gkaa1132_Supplemental_Files [file gkaa1132_supplemental_files.zip › Lavdovskaia et al Suppl data 201019.pdf]

Dual function of GTPBP6 in biogenesis and recycling of human mitochondrial ribosomes

Elena Lavdovskaia[1,3], Kärt Denks[2,3], Franziska Nadler[1], Emely Steube[1], Andreas Linden[4,5], Henning Urlaub[4,5], Marina V. Rodnina[2,3] and Ricarda Richter-Dennerlein[1,3]*

Supplementary data

Table S1: Key reagents

	SOURCE	IDENTIFIER
**Antibodies**		
Rabbit polyclonal anti-uS7m	Sigma Prestige	Cat#HPA023007
Rabbit polyclonal anti-uS14m	ProteinTech	Cat#16301-1-AP
Rabbit polyclonal anti-uS15m	ProteinTech	Cat#17006-1-AP
Rabbit polyclonal anti-bS16m	ProteinTech	Cat#16735-1-AP
Rabbit polyclonal anti-mS25	ProteinTech	Cat#15277-1-AP
Rabbit polyclonal anti-mS27	ProteinTech	Cat#17280-1-AP
Rabbit polyclonal anti-uL1m	self made	PRAB4964
Rabbit polyclonal anti-uL3m	ProteinTech	Cat#16584-1- AP
Rabbit polyclonal anti-bL12m	ProteinTech	Cat#14795-1-AP
Rabbit polyclonal anti-uL13m	ProteinTech	Cat#16241-1-AP
Rabbit polyclonal anti-bL20m	ProteinTech	Cat#16969-1-AP
Rabbit polyclonal anti-uL23m	self made	PRAB1716
Rabbit polyclonal anti-bL32m	self made	PRAB4957
Rabbit polyclonal anti-mL44	ProteinTech	Cat#16394-1-AP
Rabbit polyclonal anti-mL45	ProteinTech	Cat#15682-1-AP
Rabbit polyclonal anti-mL62	ProteinTech	Cat#10403-1-AP
Rabbit polyclonal anti-MALSU1	ProteinTech	Cat#22838-1-AP
Rabbit polyclonal anti-NSUN4	ProteinTech	Cat#16320-1-AP
Rabbit polyclonal anti-GTPBP5 (MTG2)	Sigma Prestige	Cat#HPA047379
Rabbit polyclonal anti-GTPBP7 (MTG1)	ProteinTech	Cat#13742-1-AP
Rabbit polyclonal anti-GTPBP10	Novusbio	Cat#NBP1-85055
Mouse monoclonal anti-FLAG	Sigma Prestige	Cat#F1804
Rabbit polyclonal anti-MFN2	ProteinTech	Cat#12186-1-AP
Rabbit polyclonal anti-TIM23	self made	PRAB1527
Rabbit polyclonal anti-TIM44	ProteinTech	Cat#13859-1-AP
Mouse monoclonal anti-COX1	Invitrogen	Cat#459600

Mouse monoclonal anti-COX2	Abcam	Cat#ab110258
Mouse monoclonal anti-Calnexin	Proteintech	Cat#66903-1-Ig
Mouse monoclonal anti-SDHA	Invitrogen	Cat#459200
Mouse monoclonal anti-GAPDH	Santa Cruz	Cat#sc-32233
Rabbit polyclonal anti-MTERF4	Sigma Prestige	Cat#HPA027097
**Chemicals**		
Anti-FLAG M2 Affinity Gel	Sigma-Aldrich	A2220
L-[^{35}S]methionine	Hartmann Analytic	SCM-01
EasyTides® Adenosine 5'-triphosphate, [γ-^{32}P]	PerkinElmer	BLU502Z250UC
Emetine dihydrochloride hydrate	Roth	
Lipofectamine 3000	Invitrogen	L3000-015
GeneJuice	Novagen	70967-3
Alt-R® CRISPR-Cas9 tracrRNA, ATTO™ 550	Integrated DNA technologies	1075927
Alt-R® S.p. Cas9 Nuclease V3	Integrated DNA technologies	1081058
Glutathione Sepharose™ 4B beads	GE Healthcare	17-0756-01
PreScission™ protease	GE Healthcare	27-0843-01
TRIzol® Reagent	Ambion	15596018
**Critical Commercial Assays**		
Alt-R® Genome Editing Detection Kit	Integrated DNA technologies	1075932
Rapid DNA Ligation Kit	ThermoFisher Scientific	K1423
T4 Polynucleotide Kinase (T4 PNK)	ThermoFisher Scientific	EK0031
Wizard® Plus SV Minipreps DNA Purification System	Promega	A1460
Wizard® SV Gel and PCR Clean-Up System	Promega	A9282
QuikChange Lightning Site-Directed Mutagenesis Kit	Agilent Technologies	210519-5
**Cell Lines**		
HEK293-Flp-In T-Rex	ThermoFisher Scientific	R78007
HEK293-Flp-In T-Rex-*Gtpbp6* $^{-/-}$ cl.1	This study	N/A
HEK293-Flp-In T-Rex-*Gtpbp6* $^{-/-}$ cl.2	This study	N/A
HEK293-Flp-In T-Rex-GTPBP6^{FLAG}	This study	N/A
HEK293-Flp-In T-Rex-GTPBP6$^{K187A\text{-}FLAG}$	This study	N/A
HEK293-Flp-In T-Rex-GTPBP6$^{D199A\text{-}FLAG}$	This study	N/A
HEK293-Flp-In T-Rex-GTPBP6$^{G352P\text{-}FLAG}$	This study	N/A
HEK293-Flp-In T-Rex-GTPBP6$^{S437P\text{-}FLAG}$	This study	N/A

**Oligonucleotides**		
Guide RNA: targeting the Exon 1 of GTPBP6: 5'-AGATGCGGACGAGAACGCCG-3'	This study; Integrated DNA technologies	N/A
Primer: Generation of the FLAG-tagged version of GTPBP6 Forward: 5'-TATAAAGCTTATGTGGGC CCTGCGGGCCGCCGTACGCCC-3'	This study; Microsynth	N/A
Primer: Generation of the FLAG-tagged version of GTPBP6 Reverse: 5'-TATAGATATCTTACTTGTCATCGTCGTCC TTGTAGTCTCCTGGAAAGAGCTTCCGGA ATTTGCCG-3'	This study; Microsynth	N/A
Primer: Generation of the FLAG-tagged mutant (K187A) version of GTPBP6 Forward: 5'-GCTGCCCCGACCAAG**GC**AGAACTGGAA GCCGCCTGGGGCGTG-3'	This study; Microsynth	N/A
Primer: Generation of the FLAG-tagged mutant (K187A) version of GTPBP6 Reverse: 5'-GGCGGCTTCCAGTTCT**GC**CTTGGTCGGG GCAGCCATCCTCTC-3'	This study; Microsynth	N/A
Primer: Generation of the FLAG-tagged mutant (D199A) version of GTPBP6 Forward: 5'-GGCGTGGAGGTGTTTG**C**CCGCTTCACGG TCGTCCTGCACATC-3'	This study; Microsynth	N/A
Primer: Generation of the FLAG-tagged mutant (D199A) version of GTPBP6 Reverse: 5'-GACGACCGTGAAGCGG**G**CAAACACCTCC ACGCCCCAGGCGGC-3'	This study; Microsynth	N/A
Primer: Generation of the FLAG-tagged mutant (G352P) version of GTPBP6 Forward: 5'-GTACGTGGACACCATC**CC**CTTCCTCTCC CAGCTGCCGCACGGC-3'	This study; Microsynth	N/A
Primer: Generation of the FLAG-tagged mutant (G352P) version of GTPBP6 Reverse: 5'-CAGCTGGGAGAGGAAG**GG**GATGGTGTC CACGTACAGGACGGTC-3'	This study; Microsynth	N/A
Primer: Generation of the FLAG-tagged mutant (S437P) version of GTPBP6 Forward: 5'-GAACGTCGTGCCCGTG**C**CTGCCCTGCG GGGCCACGGGCTCCAG-3'	This study; Microsynth	N/A

Primer: Generation of the FLAG-tagged mutant (S437P) version of GTPBP6 Reverse: 5’-GTGGCCCCGCAGGGCAG**G**CACGGGCACGACGTTCGGTTCCG-3’	This study; Microsynth	N/A
Probe for northern blot: targeting MTRNR1 (12S rRNA) 5’-TCGATTACAGAACAGGCTCCTCTAG-3’	This study; Microsynth	N/A
Probe for northern blot: targeting MTRNR2 (16S rRNA) 5’-GTTTGGCTAAGGTTGTCTGGTAGTA-3’	This study; Microsynth	N/A
Probe for northern blot: targeting MTCO1 5’-GTCAGTTGCCAAAGCCTCCGATTATG-3’	This study; Microsynth	N/A
Probe for northern blot: targeting MTCO2 5’-GACGTCCGGGAATTGCATCTGTTTT-3’	This study; Microsynth	N/A
Probe for northern blot: targeting 18S-rRNA 5’-TTTACTTCCTCTAGATAGTCAAGTTCGACC-3’	(1); Microsynth	
**Recombinant DNA**		
pOG44 Flp-Recombinase Expression Vector	ThermoFisher Scientific	V600520
pcDNA5/FRT/TO	ThermoFisher Scientific	V6520-20
pGex-6P-1	Merck	GE28-9546-48
**Software and Algorithms**		
ImageJ	https://imagej.nih.gov/ij/	
ImageQuant TL 7.0	GE Healthcare<br>http://www.gelifesciences.com/webapp/wcs/stores/servlet/catalog/en/GELifeSciences-de/products/AlternativeProductStructure_16016/	

Table S2: Apparent rate constant (k_{app}) of 70S ribosome recycling catalyzed by human GTPBP6 and *E.coli* RRF-EF-G (related to Figure 3)

Complex	k_{app1} (s^{-1})	k_{app2} (s^{-1})
preHC + GTPBP6	0.05 ± 0.006	
postHC + GTPBP6	0.08 ± 0.012	
70S + GTPBP6	0.09 ± 0.006	
preHC + RRF + EF-G	0.31 ± 0.05	0.02 ± 0.003
postHC + RRF + EF-G	0.34 ± 0.02	
70S + RRF + EF-G	0.33 ± 0.04	

n = 3; mean ± standard deviation.

Table S3: Label-free quantitative mass spectrometry analyses of mtLSU complexes in Gtpbp6$^{-/-}$ cells vs. WT (related to Figure 5) (see excel file)

Figure S1: Sequence alignment of human GTPBP6 and the bacterial homolog HflX from *E. coli*, *B. subtilis* and *S. aureus* using ClustalW2 (related to Figure 1).

Mutated residues analyzed in this study are indicated in red.

Figure S2: Elevated expression of GTPBP6 does not affect mtLSU assembly factors (related to Figure 2).

Mitochondrial lysates (0.5 mg) isolated from cells overexpressing $GTPBP6^{FLAG}$ were dissected by sucrose density gradient centrifugation in comparison to wild type. Fractions were analyzed by western blotting using indicated antibodies. Figure represents an extended version of Figure 1D. In addition to markers of the mtLSU and mtSSU, the levels and distribution of the assembly factors NSUN4, MALSU1 and GTPBP10 have been determined using specific antibodies.

Figure S3: 70S subunit dissociation facilitated by RRF and EF-G (related to Figure 3).

(A) Time courses of ribosome dissociation measured upon addition of RRF (2.5 µM) and EF-G (2 µM) to 70S ribosomes (0.05 µM). For method details, see Figure 3C and Methods.

(B) Dissociation of different ribosomal complexes facilitated by RRF-EF-G. Complexes were prepared as in Figure 3E. Concentrations are as in (A).

(C) Ribosome dissociation upon addition of IF3 (1 µM) to 70S ribosomes (0.05 µM). 70S + IF3: $k_{app} = 0.04\ s^{-1}$. Experiment was performed as in 3C and S3A.

Figure S4: Characterization of CRISPR/Cas9 generated *Gtpbp6* knock out cell lines (related to Figure 4).

(A) Genotyping of the $Gtpbp6^{-/-}$ clone 1 genomic DNA by Sanger sequencing. Chromatogram represents an amplicon generated by PCR covering the guide RNA target site in the first exon of GTPBP6 in $Gtpbp6^{-/-}$ clone 1. Resolving the double peaks suggests that $Gtpbp6^{-/-}$ clone 1 has heterozygous single (allele 1) or double (allele 2) nucleotide deletions.

(B) Sequence alignment of the first exons of GTPBP6-WT and *Gtpbp6*$^{-/-}$ clone 2. The absence of the double peaks in chromatogram of *Gtpbp6*$^{-/-}$ clone 2 indicates the presence of the homozygous 332 bp insertion.

(C) Schematic representation of the affected *Gtpbp6* locus (upper panel) and corresponding translation frames (lower panel). Full-length GTPBP6 consists of 516 amino acid residues. The presence of frame shift mutations leading to the premature stop codons formation in *Gtpbp6*$^{-/-}$ clone 1 results in formation of 2 truncated protein variants (113 aa – allele 1 and 102 aa – allele 2). Insertion in *Gtpbp6*$^{-/-}$ clone 2 leads to formation of 112 aa protein.

(D-E) GTPBP6^{FLAG} expression in *Gtpbp6*$^{-/-}$ cells. Expression of GTPBP6^{FLAG} in *Gtpbp6*$^{-/-}$ cells clone 1 (D) and clone 2 (E) was induced with different concentration of tetracycline as indicated. Whole cell protein extracts were isolated and analyzed by western blotting. Relative expression levels of GTPBP6^{FLAG} were determined using FLAG antibodies. Calnexin was used as a loading control.

(F) GTPBP6 loss does not affect mt-RNA levels. RNA was isolated from HEK293T-wild type (WT) and *Gtpbp6*$^{-/-}$ cells and analyzed by Northern blotting using specific probes against *MTRNR1* (12S rRNA), *MTRNR2* (16S rRNA), *MTCO1* (COX1 mRNA), *MTCO2* (COX2 mRNA) and 18S rRNA (loading control).

(G) RNA levels were visualized using Typhoon imaging system (GE Healthcare) and quantified with ImageQuant TL software. Steady state RNA levels from wild type cells are marked as dashed line (100%). (n = 3; mean ± SEM).

Figure S5. HflX binds on *E. coli* 50S subunit to the site that is also conserved in human 39S mtLSU.

(A) Superimposition of human mitochondrial 39S mtLSU (yellow, PDB 3J9M, (2)), HflX-bound *E. coli* 50S LSU (blue, PDB 5ADY, (3)) and free *E. coli* 50S LSU (green, PDB 4YBB, (4)). View from the small subunit side. HflX is shown in transparent blue.

(B) HflX contacts conserved elements of 50S subunit, such as 23S rRNA helix 69 (H69), L7/L12 stalk, sarcin-ricin loop (SRL) and peptidyl transferase center (3).

(C, D). In addition to SRL (panel B), also A- and P-loop at the PTC are structurally similar in 39S and 50S; as well as H89, which was shown to form extensive interactions with HflX on 50S subunit (3).

(E) HflX induces a displacement of H69 that forms intersubunit bridge B2a both in bacterial and mitochondrial ribosome. Its flexible loop is not resolved in 39S mtLSU. (F) Large conformational changes also occur on L7/L12 stalk of 50S upon the binding of HflX. This is illustrated with the displacement of uL11 (blue).

References

1. Larburu,N., Montellese,C., O'Donohue,M.-F., Kutay,U., Gleizes,P.-E. and Plisson-Chastang,C. (2016) Structure of a human pre-40S particle points to a role for RACK1 in the final steps of 18S rRNA processing. *Nucleic Acids Research*, **44**, 8465–8478.

2. Amunts,A., Brown,A., Toots,J., Scheres,S.H.W. and Ramakrishnan,V. (2015) Ribosome. The structure of the human mitochondrial ribosome. *Science*, **348**, 95–98.

3. Zhang,Y., Mandava,C.S., Cao,W., Li,X., Zhang,D., Li,N., Zhang,Y., Zhang,X., Qin,Y., Mi,K., *et al.* (2015) HflX is a ribosome-splitting factor rescuing stalled ribosomes under stress conditions. *Nat. Struct. Mol. Biol.*, **22**, 906–913.

4. Noeske,J., Wasserman,M.R., Terry,D.S., Altman,R.B., Blanchard,S.C. and Cate,J.H.D. (2015) High-resolution structure of the Escherichia coli ribosome. *Nat. Struct. Mol. Biol.*, **22**, 336–341.

```
GTPBP6_HUMAN    MWALRAAVRPGLRLSRVGRGRSAPRAAAPSCPARALAAVGRRSPGNLEGPWGGGRGLRAD    60
HFLX_ECOLI      ------------------------------------------------------------    0
HFLX_BACSU      ------------------------------------------------------------    0
HFLX_STAAU      ------------------------------------------------------------    0

                                             ND1 (ATPase)
GTPBP6_HUMAN    GGRSRTGDDEEEPEDADENAEEELLRGEPLLPAGTQRVCLVHPDVKWGPGKSQMTRAEWQ    120
HFLX_ECOLI      ------------MFDRYDAGEQA---------------VLVHIYF---TQDKDM----ED    26
HFLX_BACSU      ------------MNEQETIQEKA---------------ILVGCQL---PHITDE-HFENS    29
HFLX_STAAU      ----MYNMAQQQIHDTKNKLEKA---------------VLVGVHA---QDDKQF-NFEST    37
                              :     *:                  **          .:

GTPBP6_HUMAN    VAEATALVHTLDGWSVVQTMVVSTKTPDRKLIFGKGNFEHLTEKIRGSPDITCVFLNVER    180
HFLX_ECOLI      LQEFESLVS-SAGVEALQVITGSRKAPHPKYFVGEGKAVEIAEAVKATGA-SVVL-FDHA    83
HFLX_BACSU      MEELASLTK-TADGKVLTSVTQKRNRADAATYIGKGKVEELKALVEELEA-DLLI-FNDE    86
HFLX_STAAU      MEELSSLSE-TCQLEVLGQITQNRDRVDRKYYVGKGKIEEIQAFIEFKDI-DVVI-TNDE    94
                : *  :*       ..:  :. . .  .    .*:*:  .:   :.       ::   .
                                                          α-helical linker
GTPBP6_HUMAN    MAAPTKKELEAAWGVEVFDRFTVVLHIFRCNARTKEARLQVALAEMPLHRSNLKRDVAHL    240
HFLX_ECOLI      LSPAQERNLERLCECRVIDRTGLILDIFAQRARTHEGKLQVELAQLRHLATRLVRGWTHL    143
HFLX_BACSU      LSPSQLKSLATAIEVKMIDRTQLILDIFAKRARTREGKLQIELAQLQYALPRLTGQGINL    146
HFLX_STAAU      LTTAQSKSLNEALGVKIIDRTQLILEIFALRARSKEGKLQVELAQLDYLLPRLQGHGKSL    154
                ::    :.*      .::**  ::*.**  .**::*.:**: **::     .*      *

GTPBP6_HUMAN    YRGVGSRYIMGSGESFMQLQQRLLREKEAKIRKALDRLRKKRHLLRRQRTRREFPVISVV    300
HFLX_ECOLI      ERQKGGIGLRGPGETQLETDRRLLRNRIVQIQSRLERVEKQREQGRQSRIKADVPTVSLV    203
HFLX_BACSU      SRQGGGIGARGPGETKLETDRRHIRNRIHEINTQLSTVIRHRSRYRERRKKNGVLQIALV    206
HFLX_STAAU      SRLGGGIGTRGPGETKLEMDRRHIRTRMNEIKHQLRTVEEHRERYRNKRNQNQVFQVALV    214
                 *  *.    * **: :: ::* :* :  :*.  *  : .:*   *. * :  .  :::*
                                    ND2 (GTPase)
GTPBP6_HUMAN    GYTNCGKTTLIKALTGDAAIQPRDQLFATLDVTAHAGTLPSRMTVLYVDTIGFLSQLPHG    360
HFLX_ECOLI      GYTNAGKSTLFNRITEA-RVYAADQLFATLDPTLRRIDVADVGETVLADTVGFIRHLPHD    262
HFLX_BACSU      GYTNAGKSTWFNRLTSA-DSYEEDLLFATLDPMTRKMVLPSGYSVLLSDTVGFIQDLPTT    265
HFLX_STAAU      GYTNAGKSSWFNVLANE-ETYEKDQLFATLDPKTRQIQINDGFNLIISDTVGFIQKLPTT    273
                ****.**:: :: ::        * ******   :   : .    :  **:**: .**

GTPBP6_HUMAN    LIESFSATLEDVAHSDLILHVRDVSHPEAELQKCSVLSTLRGLQLPAPLLDSMVEVHNKV    420
HFLX_ECOLI      LVAAFKATLQETRQATLLLHVIDAADVRVQENIEAVNTVLEEIDAHE---IPTLLVMNKI    319
HFLX_BACSU      LIAAFRSTLEEVKEADLILHLIDSSNEDYAGHEKTVLRLLEELEADD---IPMLTAYNKR    322
HFLX_STAAU      LIAAFKSTLEEAKGADLLVHVVDSSHPEYRTQYDTVNDLIKQLDMSH---ISQIVIFNKK    330
                *: :* :**::.  : *::*: * :.     :  :*   :. ::         :   **

GTPBP6_HUMAN    DLVPG--YSP-------TEPNVVPVSALRGHGLQELKAELDAAVLKATGRQILTLRVRL-    470
HFLX_ECOLI      DMLED--FEPRIDRDEENKPNRVWLSAQTGAGIPQLFQALTERL---SG-EVAQHTLRLP    373
HFLX_BACSU      DQKLPD-FIPTAGRD------HIMVSAKFEDDAAAFKEAIQRYL---RQELLTSFEAHVP    372
HFLX_STAAU      DLCDHASNRPASDLP------NVFVSSKNDGDKLLVKTLFIDEI---K-RQLTYYDEAIA    380
                *        *            : :*:    .   .   :   :       :      :

GTPBP6_HUMAN    --AGAQLSWLYKEATVQEVDVIPEDGAADVRVIISNSAYGKFRKLFPG---------  516
HFLX_ECOLI      PQEGRLRSRFYQLQAIE-KEWMEEDGSVSLQVRMPIVDWRRLCKQEPALIDYLI---  426
HFLX_BACSU      ASEGKLLSRIKSETMVD-RFYFNEENEQ--------YDISGYVQEEQSIIGELKKYM  420
HFLX_STAAU      TNNADRLYFLKQHTLVT-ELKYDEIENV--------YRIKGFKK-------------  415
                   .     : .   :       *                   :
```

Lavdovskaia et al. Figure S1

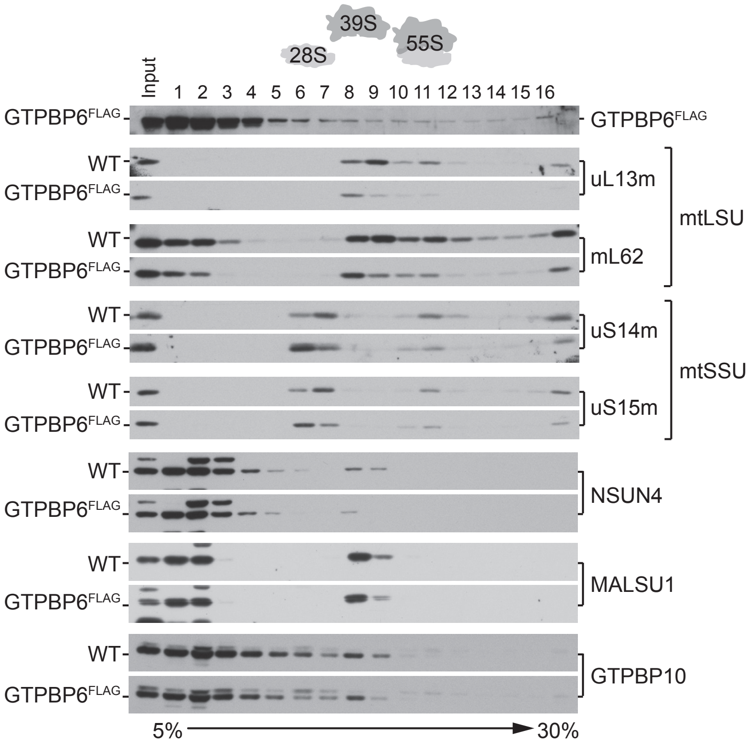

Lavdovskaia et al. Figure S2

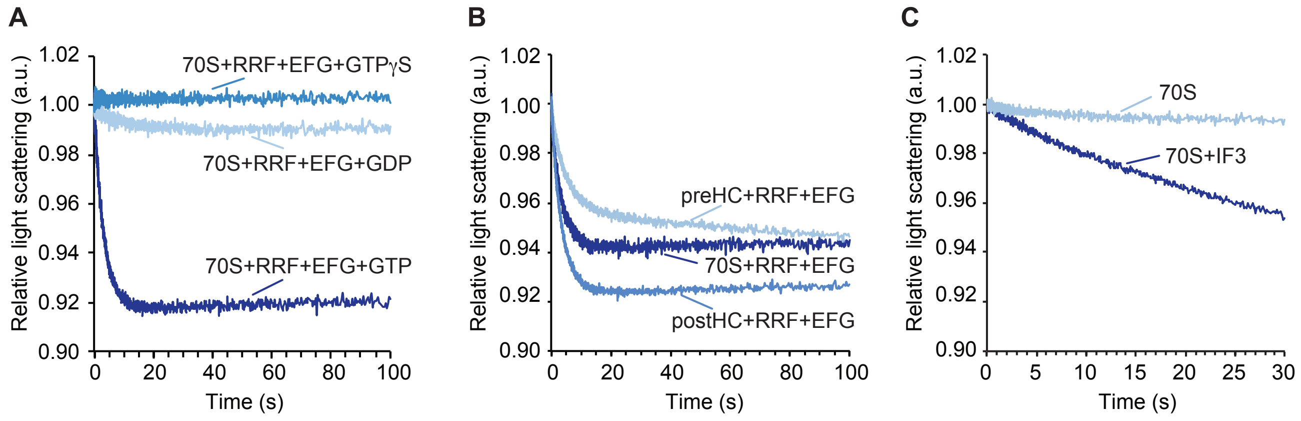

Lavdovskaia et al. Figure S3

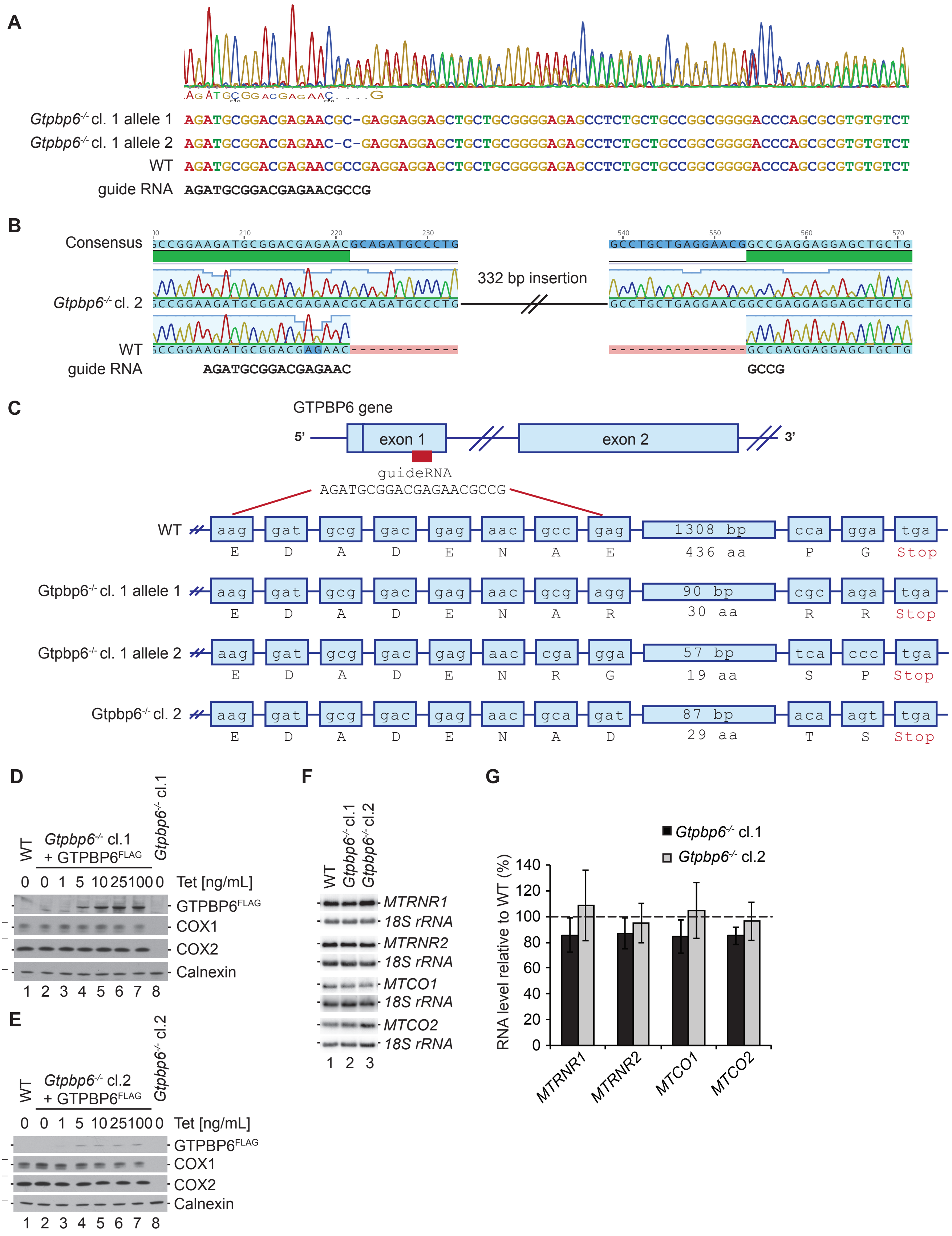

Lavdovskaia et al. Figure S4

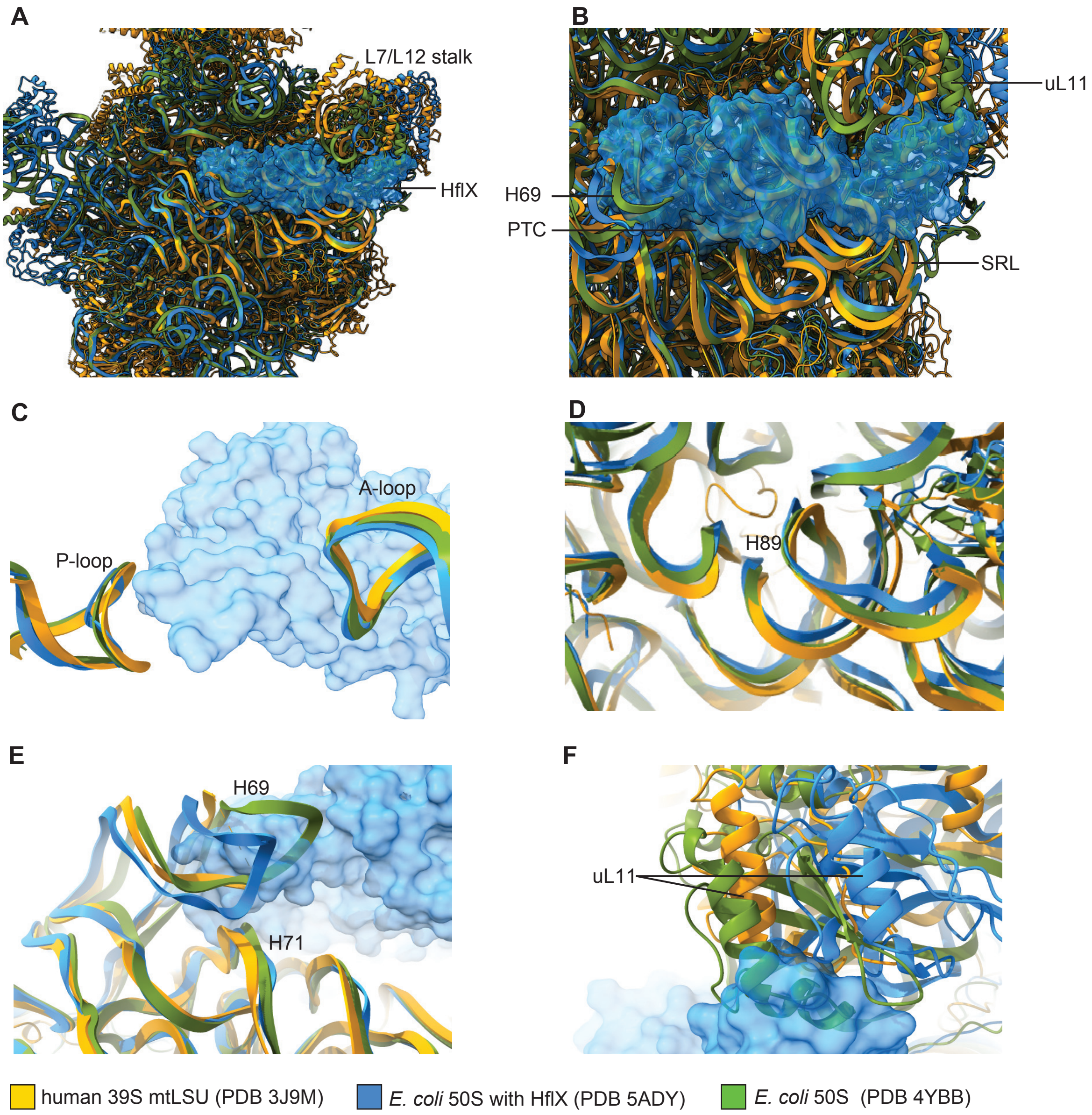

Lavdovskaia et al. Figure S5
